# Dengue severity and profiles of complement activation and immune mediators: A multicenter cohort study in Indonesia

**DOI:** 10.1371/journal.pone.0350610

**Published:** 2026-06-04

**Authors:** Ika Saptarini, Sri Masyeni, Alida Roswita Harahap, Astuti Giantini, Pringgodigdo Nugroho, Agus Handito, Harimat Hendarwan, Adityo Susilo, Sotianingsih Haryanto, Desi Fitriani, R. Tedjo Sasmono, Erni Juwita Nelwan

**Affiliations:** 1 Doctoral Program in Medical Science, Faculty of Medicine, Universitas Indonesia, Jakarta, Indonesia; 2 Research Center for Preclinical and Clinical Medicine, National Research and Innovation Agency, Cibinong, Bogor, Indonesia; 3 Department of Internal Medicine, Faculty of Medicine and Health Sciences, Universitas Warmadewa, Denpasar, Indonesia; 4 Department of Clinical Pathology, Faculty of Medicine Universitas Indonesia, Jakarta, Indonesia; 5 Division of Nephrology and Hypertension, Department of Internal Medicine, Faculty of Medicine, Universitas Indonesia, Jakarta, Indonesia; 6 Directorate General of Disease Prevention and Control, Ministry of Health of the Republic of Indonesia, Jakarta, Indonesia; 7 Division of Tropical and Infectious Disease, Department of Internal Medicine, Faculty of Medicine, Universitas Indonesia, Jakarta, Indonesia; 8 Department of Clinical Pathology, Raden Mattaher Regional General Hospital, Jambi, Indonesia; 9 Department of Internal Medicine, Khidmat Sehat Afiat Regional General Hospital, Depok, Indonesia; 10 Eijkman Research Center for Molecular Biology, National Research and Innovation Agency, Cibinong, Bogor, Indonesia; 11 Metropolitan Medical Centre Hospital, Jakarta, Indonesia; 12 Abdi Waluyo Hospital, Jakarta, Indonesia; Instituto Nacional de Salud Publica Centro de Investigaciones sobre Enfermedades Infecciosas, MEXICO

## Abstract

**Background:**

Dengue virus (DENV) infection can manifest as dengue fever (DF) or dengue hemorrhagic fever (DHF), although DHF often becomes clinically apparent around defervescence. How complement components and other immune responses evolve over the course of illness from the febrile to recovery phase remains incompletely defined. This study characterized circulating complement activation and immune mediators in DF and DHF using paired febrile and early-recovery samples.

**Methods:**

We conducted a multicenter prospective cohort study at five hospitals in Indonesia between November 2024 and October 2025. Patients with laboratory-confirmed dengue were classified as DF or DHF. Plasma concentrations of PTX3, C5a, IL-6, IL-10, IL-8, and CXCL10 were quantified in paired febrile and early recovery phase samples. Between-group differences, within-patient changes between the two time points, and correlations among immune mediators were assessed using appropriate statistical methods.

**Results:**

We included 110 confirmed dengue cases in the analysis. PTX3 and IL-10 levels were significantly higher in DHF than in DF during early recovery, whereas no mediator differed significantly between severity groups during the febrile phase. Across phases, C5a increased significantly from febrile to early recovery in DHF but not in DF, whereas PTX3 decreased significantly in DF but not in DHF. Correlations among mediators were generally weak to moderate, with a reproducible PTX3–IL-10–CXCL10 module observed across both phases.

**Conclusion:**

The measured mediators did not distinguish DF from DHF during the febrile phase, but differences emerged in early recovery, with higher PTX3 and IL-10 in DHF. Across phases, C5a increased significantly from febrile to early recovery in DHF, whereas PTX3 decreased significantly only in DF. A PTX3–IL-10–CXCL10 module was observed at both time points. Together, these patterns suggest that within-patient changes around defervescence or in the early recovery may be informative and warrant evaluation in larger, prospectively timed cohorts.

## Introduction

Dengue virus (DENV) is a mosquito-borne flavivirus responsible for dengue, one of the most widespread and clinically significant viral infections affecting humans. Globally, dengue is estimated to cause more than 400 million infections each year, resulting in approximately 22,000 deaths annually. The disease is endemic in over 100 countries across tropical and subtropical regions, where it constitutes a substantial and growing public health burden [1]. Clinically, dengue ranges from dengue fever (DF), an acute febrile illness without evidence of plasma leakage, to dengue hemorrhagic fever (DHF), in which increased vascular permeability leads to plasma leakage, as indicated by hemoconcentration or fluid accumulation [2].

A hallmark feature of DHF is a transient and reversible increase in vascular permeability, underscoring a central role for immune mediators in the disruption of endothelial homeostasis [3]. Although the mechanisms underlying severe disease are not fully defined, accumulating evidence suggests that an exaggerated host immune response drives disease progression. Heightened production of inflammatory cytokines and chemokines promotes endothelial activation and dysfunction, thereby contributing to plasma leakage [4]. Dengue is an intrinsically dynamic disease. Immune mediator levels change substantially over the course of the disease, from the febrile phase through the critical phase, early recovery, and convalescence. Prior studies show that cytokine and chemokine concentrations vary by disease phase, and that longitudinal patterns may be more informative for disease severity than single time-point measurements [5,6].

Among immune mediators implicated in dengue pathogenesis, IL-6 amplifies systemic inflammation and promotes endothelial activation, whereas IL-8 drives leukocyte recruitment, particularly neutrophil trafficking, and is also linked to endothelial activation. IL-10, a key immunoregulatory cytokine, has been consistently associated with severe disease and may represent a counter-regulatory response to excessive inflammation; however, sustained overexpression may contribute to immune dysregulation [7,8]. CXCL10 (C-X-C motif chemokine ligand 10) reflects interferon-driven immune activation and promotes recruitment of activated T cells during acute infection [5,6]. Together, these mediator pathways form an interconnected network that shapes antiviral immunity, leukocyte trafficking, and endothelial function, thereby influencing clinical severity. Growing evidence further implicates dysregulated complement activation in severe dengue through the amplification of inflammation and endothelial injury [9,10].

In addition, the complement system can limit viral replication. However, excessive activation may worsen disease severity by amplifying inflammatory responses. The anaphylatoxin C5a promotes endothelial activation, leukocyte recruitment, mast-cell degranulation, and increased vascular permeability [3,9]. Also, PTX3, a long pentraxin and regulator of complement activation, is rapidly produced by endothelial and myeloid cells in response to inflammation. PTX3 can modulate complement activity and has been linked to severe dengue and vascular involvement [11,12].

However, integrated analyses that jointly examine immune mediators, including complement components across dengue phases, using paired samples, remain limited [5,10]. Because immune activation changes rapidly around defervescence and early recovery, sampling at two clinically defined time points provides a practical way to compare phase-specific profiles and estimate within-person changes while minimizing between-patient variability. Accordingly, we measured PTX3, C5a, IL-6, IL-10, IL-8, and CXCL10 at febrile and early recovery and evaluated their associations with disease severity, to better contextualize the coordinated inflammatory, immunoregulatory, and complement-related responses during dengue illness [5,10].

## Methods

### Study design

This multicenter, prospective, observational cohort study was conducted across five hospitals in West Java, Jakarta, and Jambi, Indonesia, including Universitas Indonesia Hospital, Khidmat Sehat Afiat Regional General Hospital, Metropolitan Medical Centre (MMC) Hospital, Abdi Waluyo Hospital, and Siloam Jambi Hospital. We enrolled non-pregnant patients aged ≥15 years who presented within 5 days of symptom onset and followed them prospectively from admission through discharge. Recruitment ran from 10/11/2024–31/10/2025, but was staggered across sites: Universitas Indonesia Hospital and Khidmat Sehat Afiat Regional General Hospital began enrollment on 10/11/2024, whereas MMC Hospital, Abdi Waluyo Hospital, and Siloam Jambi Hospital started on 01/03/2025 after the amended protocol was approved by the Health Research Ethics Committee, National Research and Innovation Agency. In total, 110 patients met the eligibility criteria and were included in the analysis. The severity of dengue patients was classified into DF and DHF according to the WHO 1997 criteria [2].

Each eligible participant provided two paired blood samples. The first was collected during the febrile phase, defined as illness days 1–5. The second was collected during early recovery, characterized by rising platelet counts and clinical improvement. EDTA whole blood was collected into suitable tubes, centrifuged, aliquoted, and stored at −80 °C until analysis.

### Molecular assay (RT‐qPCR)

Viral RNA was extracted from plasma using the QIAamp Viral RNA Mini Kit (Qiagen, Hilden, Germany). DENV detection and serotype identification were carried out using the CDC real-time RT-qPCR assay for DENV-1–4, with amplification performed using the Luna One-Step RT-qPCR reagent (New England Biolabs, Ipswich, MA, USA) [13]. Appropriate positive and negative controls were included in each PCR run.

### Serological assay

Immunologic status for dengue was determined using the NovaLisa Dengue Virus IgG ELISA (NovaTec Immundiagnostica, Germany) on febrile phase sample, performed according to the manufacturer’s instructions. IgG-positive results were interpreted as indicating secondary dengue infections.

### Immune mediator measurement

Magnetic bead–based multiplex assays were performed using a premixed microparticle panel containing capture antibodies against human PTX3, C5a, IL-6, IL-10, IL-8, and CXCL10 (R&D Systems, Minneapolis, MN, USA). Diluted plasma samples, standards, and bead suspensions were incubated in microplate wells for 2 hours at room temperature with agitation, followed by washing, incubation with a biotinylated detection antibody cocktail for 1 hour, and subsequent incubation with streptavidin–phycoerythrin for 30 min. After a final wash, fluorescence was acquired using a MAGPIX system, and concentrations were interpolated from manufacturer-provided standard curves. Nine healthy controls were included in parallel to provide a reference baseline and to monitor assay performance. Values below the lower limit of quantification (LLOQ) without a quantitative estimate were imputed as LLOQ/√2, whereas values above the upper limit of quantification (ULOQ) were imputed as 1.1 × ULOQ. These imputation rules were applied consistently across analytes, in accordance with commonly used approaches for censored multiplex biomarker data [14]. The median immune mediator concentrations for the healthy control group, together with the number of imputed observations, are reported in S1 Table.

### Statistical analysis

Statistical analyses were performed using Stata/SE 15.1 (StataCorp LLC, College Station, TX, USA) and GraphPad Prism 10. The Shapiro–Wilk test is used to assess data normality. Continuous variables are presented as the mean ± SD for normally distributed data and as the median (IQR) for non-normally distributed data. Categorical variables were compared using the chi-square test.

The primary analysis was phase-based and focused on paired comparisons between the febrile and early recovery phases. Because immune mediator data were not normally distributed, comparisons between phases were performed using the Wilcoxon signed-rank test. While comparisons between dengue DF and DHF within the same clinical phase were performed using the Mann–Whitney U test.

As a supportive analysis, samples were additionally categorized by day of illness into three stages (days 1–3, 4–5, and 6–9) to describe temporal patterns, regardless of whether the sample was obtained during the febrile or early recovery phase. Not all participants contributed observations to all day-of-illness categories. Comparisons between DF and DHF within the same day-of-illness category were performed using the Mann–Whitney U test. In addition, exploratory mixed-effects models were fitted for each log-transformed immune mediator, with disease severity, clinical phase, and their interaction included as fixed effects, and participant included as a random intercept to account for within-participant correlation across repeated measurements. Associations among immune mediators were evaluated using Spearman’s rank correlation analysis. A p-value < 0.05 was considered statistically significant.

### Ethics statement

The study protocol was approved by the Health Research Ethics Committee of the National Research and Innovation Agency (Approval No. 110/KE.03/SK/05/2024, dated 30/05/2024), with an amendment approved under No. 031/KE.03/AMD/02/2025 (dated 28/02/2025). Site-specific ethical approval was obtained from Universitas Indonesia Hospital (Approval No. S-099/KETLIT/RSUI/VII/2024, dated 11/07/2024). The protocol was also approved by the Ethics Committee of the Faculty of Medicine, University of Indonesia–Cipto Mangunkusumo Hospital (Approval No. KET-1593/UN2.F1/ETIK/PPM.00.02/2024, dated 04/11/2024) and subsequently amended (No. ND-305/UN2.F1/ETIK/PPM.00.02/2025, dated 16/05/2025). Written informed consent was obtained from all participants; for adolescents, assent was obtained, along with consent from a parent or legal guardian when applicable.

## Results

Clinical and laboratory characteristics of the study participants are summarized in Table 1. A total of 110 dengue patients met the inclusion criteria and were included in the analysis. Among them, 47 participants were classified as DF and 63 as DHF. Age and sex distributions did not differ significantly between the groups (p = 0.174 and p = 0.344, respectively). Patients with DHF had a significantly longer length of hospital stay than those with DF (p = 0.001). Also, the proportion of comorbidities was higher in DHF than in DF (p = 0.004). Serotype distribution and infection type did not differ significantly between groups (p = 0.877 and p = 0.626, respectively). The day of illness at admission was also similar between groups (p = 0.432). Admission symptoms were largely similar; however, myalgia was more frequently observed in DF patients (p = 0.027). Laboratory indices at admission did not differ significantly. In contrast, DHF patients exhibited a lower nadir platelet count (p < 0.001), higher peak hematocrit (p = 0.011), elevated AST and ALT levels (p = 0.003 and p = 0.005, respectively), and lower albumin levels (p < 0.001).

**Table 1:** Demographic, clinical, and laboratory characteristics of the study participants.

Variable	DF (47)	DHF (63)	p-value§
Age (years)*	31 (22-39)	34 (25-44)	0.174
Length of hospital stay (days)*	4 (3-5)	5 (4-7)	**0.001**
Sex†			0.344
Female	23 (48.9)	36 (58.1)	
Male	24 (51.1)	26 (41.9)	
Comorbid†	4 (8.5)	20 (31.8)	**0.004**
Infecting serotype detection†			0.877
DENV-1	7 (14.9)	11 (17.5)	
DENV-2	8 (17.0)	15 (23.8)	
DENV-3	23 (48.9)	28 (44.4)	
DENV-4	8 (17.0)	8 (12.7)	
Not detected	1 (2.1)	1 (1.6)	
Type of infection†			0.626
Primary	5 (10.6)	5 (9.1)	
Secondary	42 (89.4)	58 (90.9)	
Days after the onset of fever (days)‡	3.2 (1.1)	3.1 (1.4)	0.432
Symptoms on admission†			
Fever	46 (98.4)	62 (98.2)	0.834
Retro orbital pain	23 (48.9)	27 (42.7)	0.526
Cephalgia	40 (85.1)	51 (81.0)	0.569
Myalgia	37 (78.7)	37 (58.7)	**0.027**
Arthralgia	22 (46.8)	25 (39.7)	0.455
Nausea	28 (59.6)	37 (58.7)	0.929
Vomitus	14 (29.8)	20 (31.8)	0.826
Abdominal pain	27 (57.5)	35 (55.6)	0.843
Tenderness	26 (55.3)	31 (49.2)	0.526
Bleeding	4 (8.5)	10 (15.9)	0.252
Laboratory			
Hemoglobin on admission (g/dL)‡	13.8 (1.8)	14.2 (1.8)	0.241
Hematocrit on admission (%)‡	40.7 (4.6)	41.8 (5.0)	0.255
Erythrocyte on admission (×103/μL)‡	5.0 (0.6)	4.9 (0.6)	0.575
Platelet count on admission (×103/μL)*	113 (73-149)	102 (59-141)	0.240
WBC on admission (×103/μL)*	3.3 (2.5-4.8)	3.9 (2.7-5.7)	0.096
Nadir platelet count (×103/μL)*	62 (25-104)	21 (13-36)	**<0.001**
Peak hematocrit (%)‡	42.1 (4.6)	44.7 (5.4)	0.011
AST (IU/L)*	85,0 (56,0-133,0)	135,0 (86,7-242,0)	**0,003**
ALT (IU/L)*	58,7 (37,7-108,0)	93,0 (54,0-168,0)	**0,005**
Albumin (g/dL)‡	3,7 (0,3)	3,2 (0,3)	**<0,001**

(*) median (interquartile range); (†) n (%); (‡) mean ± standard deviation; (§) Bold values denote statistical significance (p < 0.05); DF: Dengue fever; DHF: Dengue hemorrhagic fever; WBC: white blood cell count. AST: Aspartate Aminotransferase; ALT: Alanine Aminotransferase.

**Fig 1:**
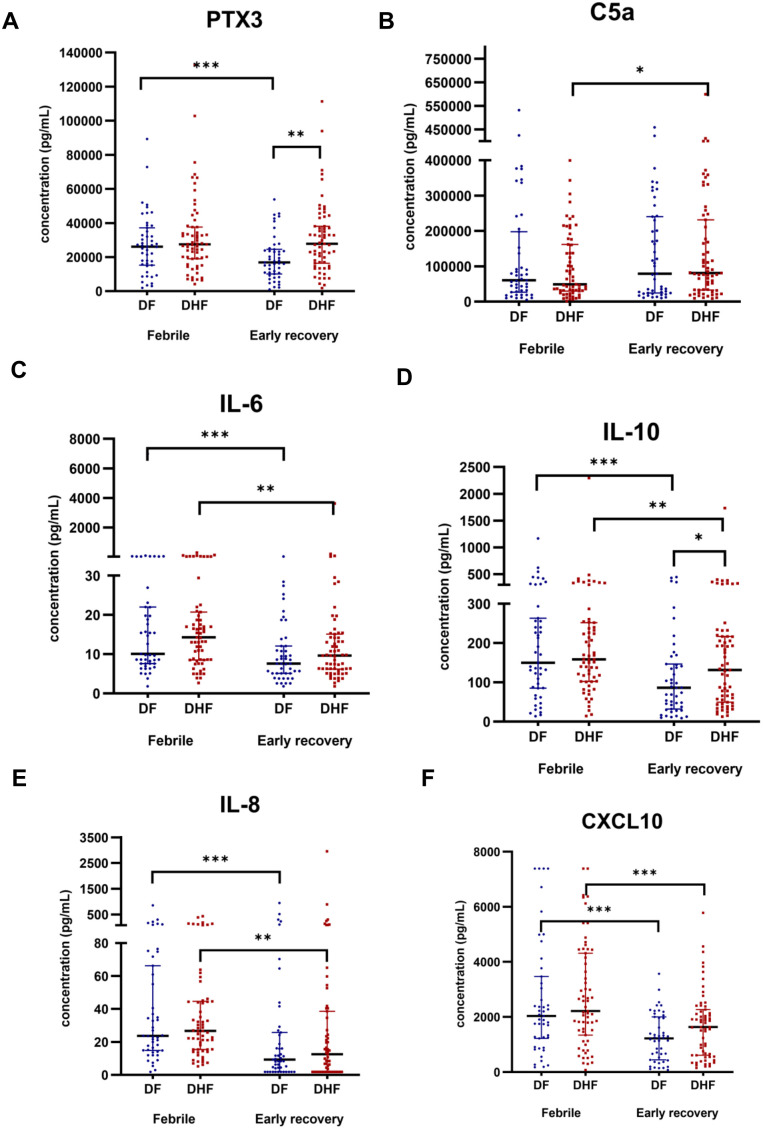
Immune mediator concentrations at two time points in dengue fever (DF) and dengue hemorrhagic fever (DHF). Plasma concentrations of **(a)** PTX3, **(b)** C5a, **(c)** IL-6, **(d)** IL-10, **(e)** IL-8 and **(f)** CXCL10 are shown at febrile and early recovery**.** Each dot represents an individual participant; horizontal bars indicate the median (IQR). Comparisons between DF and DHF within each time point were performed using the Mann–Whitney U test, whereas within-participant comparisons between the febrile and early recovery phases were performed using the Wilcoxon signed-rank test. Statistical significance is indicated for comparisons between DF and DHF within each time point and, where shown, between time points. *p < 0.05; **p < 0.01; ***p < 0.001.

Plasma concentrations of PTX3, C5a, IL-6, IL-10, IL-8, and CXCL10 were compared between patients with DF and DHF during the febrile and early recovery phases (Fig 1). During the febrile phase, no significant differences were observed between DF and DHF (p > 0.05). In contrast, during the early recovery phase, PTX3 and IL-10 levels were significantly higher in DHF than in DF (p < 0.01 and p < 0.05, respectively), whereas C5a, IL-6, IL-8, and CXCL10 levels did not differ significantly between severity groups. Paired analyses demonstrated significant temporal changes from the febrile to early recovery phase, with IL-6, IL-10, IL-8, and CXCL10 levels declining significantly over time in both DF and DHF groups. In contrast, PTX3 levels declined significantly from the febrile to the early recovery phase in DF (p < 0.001), whereas no significant change was observed in DHF. Similarly, C5a levels did not change significantly across phases in DF but increased significantly in DHF (p < 0.05). Median values (interquartile ranges) of mediator concentrations in DF and DHF during the febrile and early recovery phases, along with the p-values for the appropriate statistical tests, are shown in S2 and S3 Tables.

Because secondary dengue predominated in our cohort, we performed a sensitivity analysis restricted to participants with secondary dengue infection. The overall phase-related patterns across the measured immune mediators were broadly consistent with those observed in the full cohort. The results of the analysis are shown in S4 and S5 Tables.

Exploratory mixed-effects analyses, using DF and the febrile phase as the reference categories, are presented in S6 Table. Overall, the findings are broadly consistent with the primary analyses, especially for PTX3 and, to a lesser extent, IL-10. PTX3 showed a significant severity-by-phase interaction (β = 0.311, 95% CI 0.056 to 0.565; p = 0.017), indicating that phase-related changes differ between DF and DHF. IL-10 displayed a similar directional pattern, although the interaction did not reach statistical significance (β = 0.331, 95% CI −0.031 to 0.693; p = 0.073). No significant severity-by-phase interactions were observed for C5a, IL-6, IL-8, or CXCL10.

**Fig 2:**
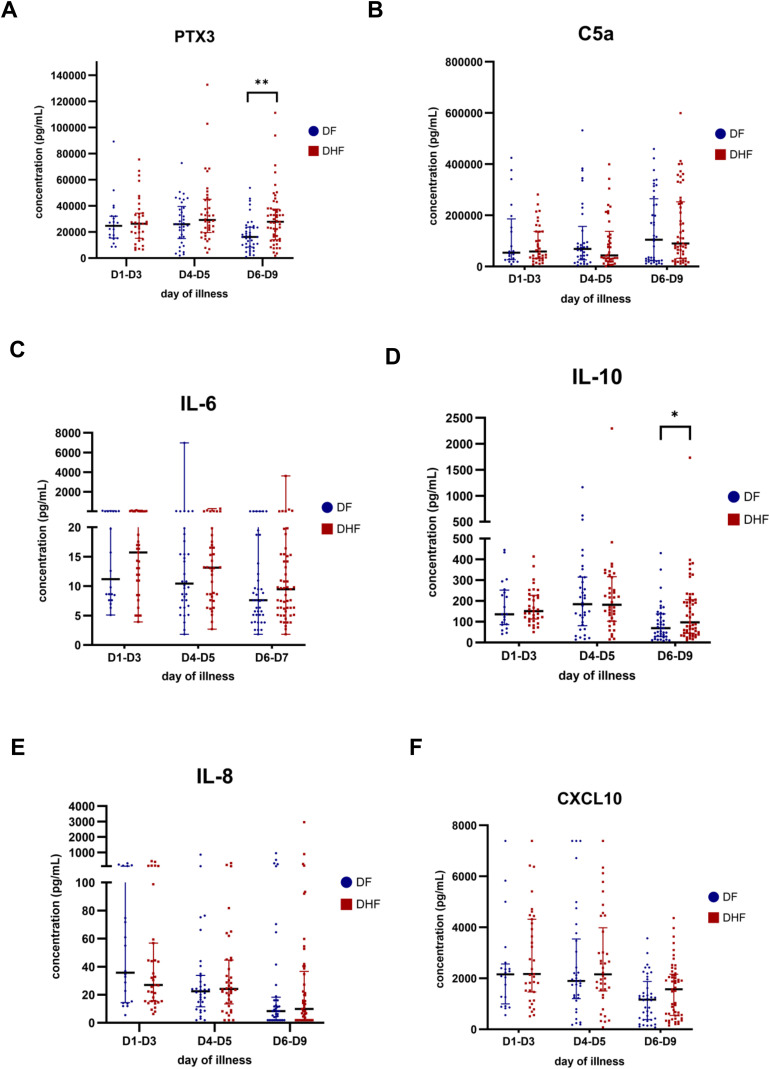
Comparable immune mediator concentrations between DF (n = 47) and DHF (n = 63) samples by day of illness. **(a)** PTX3, **(b)** C5a, **(c)** IL-6, **(d)** IL-10, **(e)** IL-8, and **(f)** CXCL10. Each dot represents the mediator level for an individual sample. Differences between DF and DHF samples within each group and on each day of illness were assessed using the Mann–Whitney test. Median values and interquartile ranges are shown for each group. Horizontal bars indicate the median, and error bars represent the interquartile range. Asterisks denote statistical significance (*p < 0.05; **p < 0.01; ***p < 0.001). DF, dengue fever; DHF, dengue hemorrhagic fever.

As an additional exploratory analysis, immune mediator concentrations were examined by day of illness to assess temporal differences between DF and DHF (Fig 2). During days 1–3 and 4–5 of illness, no significant differences were found between DF and DHF in PTX3, C5a, IL-6, IL-10, IL-8, or CXCL10 levels. In contrast, during days 6–9, significant differences were identified for selected mediators. PTX3 and IL-10 levels were significantly higher in patients with DHF than in those with DF (p < 0.01 and p < 0.05, respectively). No significant differences were observed for C5a, IL-6, IL-8, or CXCL10 during this period. The median values (interquartile ranges) and p values of mediator concentrations stratified by day of illness and dengue severity are presented in S7 Table**.**

**Fig 3:**
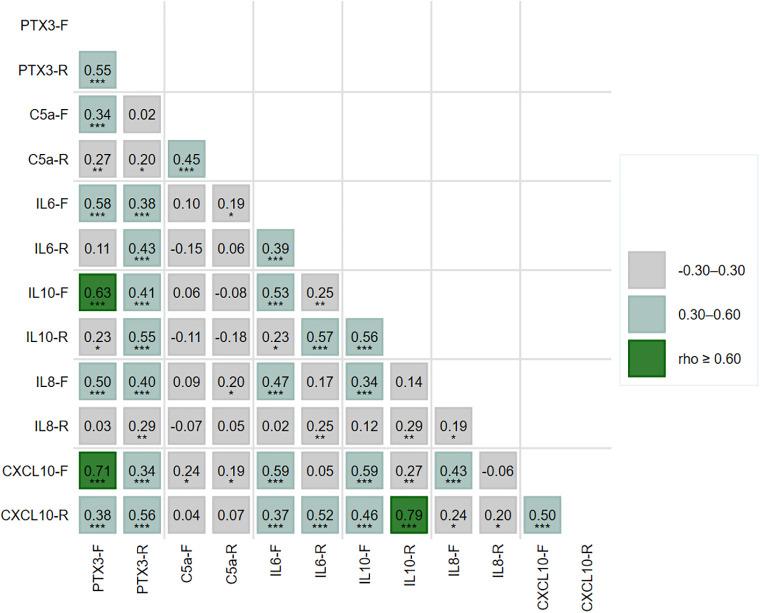
Spearman correlation matrix of immune mediators measured in paired febrile and early recovery dengue samples. Cells display Spearman’s ρ values for pairwise correlations among PTX3, C5a, IL-6, IL-10, IL-8, and CXCL10 measured in febrile (F) and early recovery (R) samples; only the lower triangle is shown. Shading indicates the magnitude of correlation (|ρ| < 0.30, 0.30–0.60, ≥ 0.60). Asterisks denote statistical significance (*p < 0.05; **p < 0.01; ***p < 0.001).

To characterize coordination among immune mediators across illness phases, we assessed pairwise associations using Spearman’s rank correlation (Fig 3). Overall, correlations were predominantly positive and were generally weak to moderate in magnitude, with strong relationships observed for only a limited subset of immune mediator pairs. Within-participant cross-phase concordance was moderate for PTX3 (ρ = 0.55), C5a (ρ = 0.45), IL-6 (ρ = 0.39), IL-10 (ρ = 0.56), and CXCL10 (ρ = 0.50). In contrast, IL-8 exhibited weak cross-phase concordance (ρ = 0.19), suggesting greater temporal variability.

During the febrile phase, a coherent PTX3–IL-10–CXCL10 module was apparent, with strong correlations between PTX3 and CXCL10 (ρ = 0.71) and between PTX3 and IL-10 (ρ = 0.63), as well as a moderate correlation between IL-10 and CXCL10 (ρ = 0.59). IL-6 showed moderate correlations with CXCL10 (ρ = 0.59), PTX3 (ρ = 0.58), IL-10 (ρ = 0.53), and IL-8 (ρ = 0.47). In contrast, C5a exhibited limited connectivity, displaying only weak-to-moderate correlations with PTX3 (ρ = 0.34) and CXCL10 (ρ = 0.24).

During early recovery, the PTX3–IL-10–CXCL10 module persisted and further strengthened, most notably between IL-10 and CXCL10 (ρ = 0.79), along with moderate correlations of PTX3 with IL-10 (ρ = 0.55) and CXCL10 (ρ = 0.56). IL-6 remained moderately correlated with IL-10 (ρ = 0.57), CXCL10 (ρ = 0.52), and PTX3 (ρ = 0.43). IL-8 continued to show weaker associations with PTX3 and IL-6 (ρ = 0.20 and ρ = 0.29, respectively). C5a remained weakly correlated with PTX3 (ρ = 0.20).

## Discussion

We characterized the dynamics of immune mediators, including complement components, across disease phases and severity. Baseline clinical characteristics were broadly similar between DF and DHF, including age, sex distribution, day of illness at presentation, and serotype, as well as most symptoms at admission, thereby minimizing potential confounding by demographic or timing-related factors. As expected, patients with DHF had longer hospital stays, a higher proportion of comorbidities, lower nadir platelet counts, and higher peak hematocrit levels. These findings are consistent with some previous studies that reflect greater plasma leakage and increased disease severity [15,16].

In our cohort, DHF was associated with higher AST and ALT levels, reflecting greater hepatic involvement. This finding may result from a combination of direct and immune-mediated hepatocellular injury, and systemic hypoperfusion [17]. In parallel, lower albumin levels in DHF are biologically plausible, as DHF is characterized by increased vascular permeability and plasma leakage, and hypoalbuminemia is recognized as one manifestation of this process. Accordingly, lower albumin levels in DHF are plausibly explained by albumin extravasation and fluid shifts [18].

Our study found that the distribution of primary and secondary dengue infections did not differ significantly between DF and DHF. This may reflect the hyperendemic setting in Indonesia, where multiple DENV serotypes co-circulate, and prior exposure is common, with secondary infection frequently observed among hospitalized dengue patients. [19]. This predominance may reduce observable differences in the proportions of primary and secondary infections between DF and DHF, even though heterologous secondary infection is associated with an increased risk of severe dengue [20,21].

Our results highlight the dynamic, phase-dependent expression of immune mediators in dengue. No immune mediator differed significantly between DF and DHF during the febrile phase or during illness days 1–3 and 4–5. This finding may indicate that this phase reflects a more generalized inflammatory response to infection, before clearer separation by disease severity becomes apparent [5,22]. By contrast, PTX3 and IL-10 were higher in DHF during early recovery and on illness days 6–9. This finding indicates that severity-related differences became more apparent later in the clinical course. This later separation may reflect slower resolution or more persistent elevation of some immune mediators around the critical and early recovery period in more severe dengue [5,23].

PTX3 is a soluble pattern-recognition molecule rapidly induced at inflammatory sites by myeloid cells and activated endothelium, mainly in response to IL-1β and TNF-α [24]. Beyond serving as an inflammatory marker, PTX3 can modulate complement, placing it at the interface of innate immunity, vascular inflammation, and complement regulation [25]. This is relevant in dengue, particularly DHF, where dysregulated innate immune responses may contribute to endothelial injury, glycocalyx disruption, and plasma leakage around the critical period near defervescence [26]. In keeping with this, previous studies have shown higher PTX3 levels in severe dengue, supporting its relevance to disease severity [11].

Also, IL-10 is an immunoregulatory cytokine that is frequently elevated alongside inflammatory activation in dengue. IL-10 has repeatedly been associated with more severe clinical outcomes, although effect sizes vary widely depending on sampling time and case definitions [5,27]. In our cohort, IL-10 did not differ significantly between DF and DHF during the febrile phase, whereas higher levels were observed in DHF during early recovery. This pattern suggests that severity-related divergence in IL-10 became more apparent later in the clinical course rather than during the febrile phase. Previous studies have also linked IL-10 to severe dengue, while showing that its levels may vary across illness phases and may remain higher later in more severe disease [23,27].

Our study found that most immune mediator levels declined from the febrile to the early recovery phase, consistent with temporal changes in host immune mediator profiles across the course of dengue illness [5]. However, the magnitude and persistence of this decline varied depending on the specific immune mediator and disease severity [5,28]. Our findings on the changes of PTX3 and C5a provide important insights into the roles of innate immune and complement system activation pathways in dengue pathogenesis. PTX3 levels did not differ significantly between the febrile and early recovery phases in the DHF group, reflecting persistence of PTX3 in the DHF group [12,29].

C5a increased significantly from the febrile to the early recovery phase in DHF, but not in DF. Among complement mediators, C5a is a potent inflammatory effector that can amplify leukocyte recruitment, endothelial activation, and vascular permeability. Excessive complement activation has been implicated in dengue immunopathogenesis [30,31]. This is consistent with evidence linking complement activation to dengue pathogenesis and endothelial dysfunction, including increased alternative pathway activity during DENV infection [32,33]. Persistently elevated C5a has also been reported after severe COVID-19, including after hospital discharge, supporting the biological plausibility that increased C5a may extend beyond the acute phase in severe inflammatory disease [34].

The timing of sample collection may have influenced our findings. In dengue, plasma leakage typically occurs around defervescence and is confined to a brief critical phase. Some samples classified as early recovery in DHF may therefore have been collected close to this window, potentially reducing the apparent separation between febrile and early-recovery measurements. This may partly account for the limited phase-to-phase change in PTX3, in keeping with previous reports linking higher PTX3 levels to severe dengue. By contrast, CXCL10, IL-6, and IL-8 declined over time but did not consistently distinguish DF from DHF, underscoring the time-dependent nature of cytokine and chemokine profiles in dengue and the variability of their association with severity across studies [11,22].

We found that inter-phase correlations for the same mediators were moderate for PTX3, C5a, IL-6, IL-10, and CXCL10, suggesting partial within-person rank stability across phases. In contrast, IL-8 showed a weak cross-phase association, plausibly reflecting its tightly regulated and rapidly inducible biology, as well as strong timing dependence relative to the critical phase window [35,36].

Regarding intra-phase correlations, immune mediator networks were predominantly positive and exhibited a weak-to-moderate correlation structure, with a reproducible PTX3, IL-10, and CXCL10 module observed across both phases. This cluster is plausible, as PTX3 is a pattern-recognition molecule linked to inflammation and endothelial activation and can also interface with complement regulation, providing a mechanistic bridge between innate sensing, vascular inflammation, and complement control [37]. In dengue, higher PTX3 levels have been reported in severe cases [11]. Meanwhile, IL-10 is frequently interpreted as part of a compensatory counter-regulatory response and has been reported at higher levels in more severe dengue, which could contribute to its co-variation with CXCL10 [38]. CXCL10 is an interferon-inducible chemokine that signals via CXCR3 and is commonly elevated in viral infections [39]. CXCL10 has been reported to track with dengue clinical severity or key manifestations in several cohorts, although the strength and timing of associations vary by study design and sampling window [40,41].

In conclusion, our findings suggest that dengue severity may reflect not only early inflammatory activation but also phase-dependent immune changes, particularly in DHF, including changes in complement-related markers. Inter-phase correlations showed moderate within-person rank stability for PTX3, C5a, IL-6, IL-10, and CXCL10, while intra-phase correlations were predominantly positive and weak to moderate, with a reproducible PTX3–IL-10–CXCL10 module across phases.

Key strengths of this study include its multicenter prospective design and paired febrile and early-recovery samples collected during hospitalization, which enabled assessment of within-patient immune changes rather than reliance on cross-sectional comparisons alone. In addition, integrated profiling of cytokines, chemokines, and complement components provided a broader view of coordinated host-response patterns. Several limitations should also be considered. The moderate sample size and limited sampling time points may have reduced sensitivity to subtler temporal patterns, and the small number of primary infections precluded robust stratified analyses by infection status. Larger longitudinal studies with denser sampling are needed to refine composite immune mediator signatures and improve risk stratification in dengue.

## Supporting information

S1 Table Healthy control concentrations and out-of-range measurements. (DOCX)

S2 Table Immune mediator concentrations in DF and DHF during the febrile and early recovery phases. (DOCX)

S3 Table Changes in immune mediator concentrations from the febrile to the early recovery phase in DF and DHF. (DOCX)

S4 Table Immune mediator concentrations in DF and DHF among participants with secondary dengue infection. (DOCX)

S5 Table Changes in immune mediator concentrations from the febrile to the early recovery phase among participants with secondary dengue infection. (DOCX)

S6 Table Exploratory mixed-effects models of immune mediators by disease severity and clinical phase. (DOCX)

S7 Table Immune mediator concentrations by day of illness and dengue severity. (DOCX)
