# Supplementary material for: Dengue severity and profiles of complement activation and immune mediators: A multicenter cohort study in Indonesia
Source: PLoS One. 2026 Jun 4;21(6):e0350610. doi: 10.1371/journal.pone.0350610 (PMC13235920; doi:10.1371/journal.pone.0350610)
Supplement: S1 Table — (DOCX) [file pone.0350610.s001.docx]

**S1 Table. Healthy control concentrations and out-of-range measurements.**

| **Mediator** | **Health control (pg/mL)** | **<LLOQ for Health control (n)** | **DF-DHF** | |
| --- | --- | --- | --- | --- |
|  |  |  | **<LLOQ (n)** | **>ULOQ (n)** |
| PTX3 | 1,287 (1,287-1,287) | 8 | 0 | 0 |
| C5a | 14,445 (7,969-34,949) | 0 | 0 | 0 |
| IL-6 | 1.9 (1.8-2.2) | 7 | 3 | 0 |
| IL-10 | 2.8 (2.0-6.6) | 7 | 0 | 0 |
| IL-8 | 8.8 (5.0-9.6) | 0 | 6 | 0 |
| CXCL-10 | 20.5 (17.9-24.8) | 0 | 0 | 6 |

Healthy-control immune mediator concentrations are presented as median (interquartile range) in pg/mL. The table also summarizes the number of samples with values below the lower limit of quantification (LLOQ) or above the upper limit of quantification (ULOQ) in healthy controls and across dengue patient samples (DF/DHF). LLOQ, lower limit of quantification; ULOQ, upper limit of quantification; DF: dengue fever; DHF: dengue hemorrhagic fever
