# Supplementary material for: Dengue severity and profiles of complement activation and immune mediators: A multicenter cohort study in Indonesia
Source: PLoS One. 2026 Jun 4;21(6):e0350610. doi: 10.1371/journal.pone.0350610 (PMC13235920; doi:10.1371/journal.pone.0350610)
Supplement: S3 Table — (DOCX) [file pone.0350610.s003.docx]

**S3 Table. Changes in immune mediator concentrations from the febrile to the early recovery phase in DF and DHF.**

| **Mediator** | | **Febrile** | **Early recovery** | **p-value** |
| --- | --- | --- | --- | --- |
| **DF** |  |  |  |  |
|  | PTX3 | 26,024 (15,233-37,159) | 16,909 (10,076- 24,510) | <0.001 |
|  | C5a | 60,295 (26,645-197,601) | 79,197 (24,195-240,378) | 0.267 |
|  | IL-6 | 10.1 (7.6- 22.0) | 7.6 (5.1-12.1) | <0.001 |
|  | IL-10 | 150.1 (85.2-263.6) | 86.2 (31.9-147.0) | <0.001 |
|  | IL-8 | 23.7 (14.8-66.2) | 9.3 (1.8-25.8) | 0.007 |
|  | CXCL-10 | 2,037 (1,226-3471) | 1,221 (446-2,001) | <0.001 |
| **DHF** | |  |  |  |
|  | PTX3 | 27,436 (18,950-37,613) | 27,847 (16,441-38,065) | 0.978 |
|  | C5a | 48,953 (29,502-162,160) | 80,720 (32,563-231,275) | 0.033 |
|  | IL-10 | 158.4 (102.3-252.3) | 131.6 (49.3-217.1) | 0.007 |
|  | IL-6 | 14.3 (8.5- 20.7) | 9.6 (6.2-15.1) | 0.002 |
|  | IL-8 | 26.7 (15.4-44.6) | 12.5 (1.8-38.6) | 0.004 |
|  | CXCL-10 | 2,219 (1,345-4,315) | 1,638 (609-2,271) | <0.001 |

Immune mediator concentrations are presented as medians (interquartile range). P-values are from paired febrile and early recovery measurements within each severity group (DF and DHF), using the Wilcoxon signed-rank test. Units are pg/mL. DF: dengue fever; DHF: dengue hemorrhagic fever.
