# Supplementary material for: Dengue severity and profiles of complement activation and immune mediators: A multicenter cohort study in Indonesia
Source: PLoS One. 2026 Jun 4;21(6):e0350610. doi: 10.1371/journal.pone.0350610 (PMC13235920; doi:10.1371/journal.pone.0350610)
Supplement: S5 Table — (DOCX) [file pone.0350610.s005.docx]

**S5 Table. Changes in immune mediator concentrations from the febrile to the early recovery phase among participants with secondary dengue infection.**

| **Mediator** | | **Febrile** | **Early recovery** | **p-value** |
| --- | --- | --- | --- | --- |
|  |  | **Median (IQR)** | **Median (IQR)** |  |
| **DF** |  |  |  |  |
|  | PTX3 | 26,892 (15,233-38,496) | 17,246 (10,076-25,187) | 0.001 |
|  | C5a | 57,403 (26,645-205,989) | 104,839 (26,690-240,378) | 0.223 |
|  | IL-6 | 11.4 (7.6-22.0) | 7.5 (5.1-12.1) | <0.001 |
|  | IL-10 | 145.1 (85.2-293.8) | 71.5 (29.9-141.5) | <0.001 |
|  | IL-8 | 23.1 (14.1- 60.9) | 8.3 (1.8-25.8) | 0.007 |
|  | CXCL-10 | 2,144 (1,261-3,471) | 1167 (446-1166) | <0.001 |
| **DHF** | |  |  |  |
|  | PTX3 | 27,511 (18,950-37,613) | 27,544 (16,441-38,060) | 0.824 |
|  | C5a | 47,726 (24,564-160,376) | 81,298 (31,438-211,029) | 0.021 |
|  | IL-10 | 14.8 (8.6-19.8) | 9.7 (6.3-15.1) | 0.002 |
|  | IL-6 | 158.9 (102.3-253.0) | 131.9 (49.3-217.1) | 0.005 |
|  | IL-8 | 26.2 (15.4-44.4) | 11.6 (1.8-38.6) | 0.003 |
|  | CXCL-10 | 2,288 (1,345-4,158) | 1,725 (608-2,271) | <0.001 |

Immune mediator concentrations are presented as medians (interquartile range). P-values are from paired febrile and early recovery measurements within each severity group (DF and DHF), using the Wilcoxon signed-rank test. Units are pg/mL. DF: dengue fever; DHF: dengue hemorrhagic fever.
