# Supplementary material for: Dengue severity and profiles of complement activation and immune mediators: A multicenter cohort study in Indonesia
Source: PLoS One. 2026 Jun 4;21(6):e0350610. doi: 10.1371/journal.pone.0350610 (PMC13235920; doi:10.1371/journal.pone.0350610)
Supplement: S6 Table — (DOCX) [file pone.0350610.s006.docx]

**S6 Table. Exploratory mixed-effects models of immune mediators by disease severity and clinical phase.**

| **Mediator** | **Fixed effect** | **β coefficient** | **95% CI lower** | **95% CI upper** | **p-value** |
| --- | --- | --- | --- | --- | --- |
| **PTX3** | DHF vs DF | 0.195 | -0.102 | 0.491 | 0.198 |
|  | Early recovery vs febrile | -0.354 | -0.547 | -0.161 | <0.001 |
|  | DHF × early recovery | 0.311 | 0.056 | 0.565 | 0.017 |
| **C5a** | DHF vs DF | -0.116 | -0.560 | 0.328 | 0.609 |
|  | Early recovery vs febrile | 0.134 | -0.223 | 0.491 | 0.463 |
|  | DHF × early recovery | 0.211 | -0.261 | 0.683 | 0.381 |
| **IL-6** | DHF vs DF | 0.022 | -0.352 | 0.396 | 0.909 |
|  | Early recovery vs febrile | -0.617 | -0.945 | -0.289 | <0.001 |
|  | DHF × early recovery | 0.277 | -0.156 | 0.710 | 0.21 |
| **IL-10** | DHF vs DF | 0.098 | -0.259 | 0.455 | 0.591 |
|  | Early recovery vs febrile | -0.661 | -0.935 | -0.387 | <0.001 |
|  | DHF × early recovery | 0.331 | -0.031 | 0.693 | 0.073 |
| **IL-8** | DHF vs DF | -0.004 | -0.528 | 0.520 | 0.988 |
|  | Early recovery vs febrile | -0.976 | -1.472 | -0.481 | <0.001 |
|  | DHF × early recovery | 0.074 | -0.580 | 0.729 | 0.824 |
| **CXCL10** | DHF vs DF | 0.108 | -0.244 | 0.460 | 0.547 |
|  | Early recovery vs febrile | -0.715 | -0.952 | -0.479 | <0.001 |
|  | DHF × early recovery | 0.223 | -0.089 | 0.535 | 0.161 |

Fixed effects from mixed-effects models are shown for each immune mediator, with disease severity (DHF vs DF), clinical phase (early recovery vs febrile), and their interaction (DHF × early recovery) included as fixed effects. Participant was included as a random intercept. DF and febrile phase were used as the reference categories.
