# Supplementary material for: Dengue severity and profiles of complement activation and immune mediators: A multicenter cohort study in Indonesia
Source: PLoS One. 2026 Jun 4;21(6):e0350610. doi: 10.1371/journal.pone.0350610 (PMC13235920; doi:10.1371/journal.pone.0350610)
Supplement: S7 Table — (DOCX) [file pone.0350610.s007.docx]

**S7 Table. Immune mediator concentrations by day of illness and dengue severity.**

| **Biomarker** | | **DF** | | **DHF** | | **p-value** |
| --- | --- | --- | --- | --- | --- | --- |
|  |  | **n** | **Median (IQR)** | **n** | **Median (IQR)** |  |
| **Day 1-3** | |  |  |  |  |  |
|  | PTX3 | 20 | 24,710 (15,338-32,108) | 35 | 26,365 (15,143-34,395) | 0.788 |
|  | C5a | 20 | 54,534 (27,173-186,473) | 35 | 59,082 (31,851-137,271) | 0.883 |
|  | IL-6 | 20 | 11.2 (7.82-31.3) | 35 | 15.7 (8.5-22.6) | 0.745 |
|  | IL-8 | 20 | 35.7 (14.4-104.7) | 35 | 26.9 (15.4-56.8) | 0.474 |
|  | IL-10 | 20 | 136.0 (85.8-251.9) | 35 | 150.6 (113.3-226.6) | 0.788 |
|  | CXCL-10 | 20 | 2,154 (997-2,562) | 35 | 2,168 (1,464-4,315) | 0.452 |
| **Day 4-5** | |  |  |  |  |  |
|  | PTX3 | 34 | 25,983 (14,891-39,537) | 38 | 29,140 (19,545-45,059) | 0.259 |
|  | C5a | 34 | 69,132 (26,559-156,738) | 38 | 43,985 (22,081-137,637) | 0.592 |
|  | IL-6 | 34 | 10.4 (6.6-17.9) | 38 | 13.1 (8.1-17.8) | 0.488 |
|  | IL-8 | 34 | 22.4 (11.3-33.8) | 38 | 24.1 (13.5-44.7) | 0.499 |
|  | IL-10 | 34 | 184.0 (80.2-314.6) | 38 | 181.9 (102.1-316.9) | 0.895 |
|  | CXCL-10 | 34 | 1,899 (1,200-3,545) | 38 | 2,160 (1,503-3,985) | 0.513 |
| **Day 6-9** | |  |  |  |  |  |
|  | PTX3 | 40 | 16,156 (8,413-23,740) | 53 | 27,847 (14,056-37,526) | 0.001 |
|  | C5a | 40 | 104,839 (22,919-264,879) | 53 | 90,404 (32,000-253,146) | 0.601 |
|  | IL-6 | 40 | 7.6 (5.1-13.4) | 53 | 9.5 (5.4-15.2) | 0.336 |
|  | IL-8 | 40 | 8.3 (1.8-18.2) | 53 | 9.9 (1.8-36.7) | 0.816 |
|  | IL-10 | 40 | 68.9 (28.9-137.0) | 53 | 96.7 (44.3-205.8) | 0.029 |
|  | CXCL-10 | 40 | 1,167 (385-1,875) | 53 | 1,572 (544-2,166) | 0.151 |

Immune mediator concentrations are shown as medians (interquartile range) for each day-of-illness window (days 1–3, 4–5, and 6–9). P-values are from Mann–Whitney tests comparing DF and DHF within each time window. Units are pg/mL. DF: dengue fever; DHF: dengue hemorrhagic fever.
